# 3D Culture of Chondrocytes in Gelatin Hydrogels with Different Stiffness

**DOI:** 10.3390/polym8080269

**Published:** 2016-07-26

**Authors:** Xiaomeng Li, Shangwu Chen, Jingchao Li, Xinlong Wang, Jing Zhang, Naoki Kawazoe, Guoping Chen

**Affiliations:** 1 International Center for Materials Nanoarchitectonics, National Institute for Materials Science, 1-1 Namiki, Tsukuba, Ibaraki 305-0044, Japan; LI.Xiaomeng@nims.go.jp (X.L.); shangwu.chan@gmail.com (S.C.); LI.Jingchao@nims.go.jp (J.L.); WANG.Xinlong@nims.go.jp (X.W.); ZHANG.Jing@nims.go.jp (J.Z.); KAWAZOE.Naoki@nims.go.jp (N.K.); 2 Department of Materials Science and Engineering, Graduate School of Pure and Applied Sciences, University of Tsukuba, 1-1-1 Tennodai, Tsukuba, Ibaraki 305-8577, Japan

**Keywords:** gelatin, hydrogel, stiffness, chondrogenic phenotype, tissue engineering

## Abstract

Gelatin hydrogels can mimic the microenvironments of natural tissues and encapsulate cells homogeneously, which makes them attractive for cartilage tissue engineering. Both the mechanical and biochemical properties of hydrogels can affect the phenotype of chondrocytes. However, the influence of each property on chondrocyte phenotype is unclear due to the difficulty in separating the roles of these properties. In this study, we aimed to study the influence of hydrogel stiffness on chondrocyte phenotype while excluding the role of biochemical factors, such as adhesion site density in the hydrogels. By altering the degree of methacryloyl functionalization, gelatin hydrogels with different stiffnesses of 3.8, 17.1, and 29.9 kPa Young’s modulus were prepared from the same concentration of gelatin methacryloyl (GelMA) macromers. Bovine articular chondrocytes were encapsulated in the hydrogels and cultured for 14 days. The influence of hydrogel stiffness on the cell behaviors including cell viability, cell morphology, and maintenance of chondrogenic phenotype was evaluated. GelMA hydrogels with high stiffness (29.9 kPa) showed the best results on maintaining chondrogenic phenotype. These results will be useful for the design and preparation of scaffolds for cartilage tissue engineering.

## 1. Introduction

Cartilage defects are common diseases in our daily life, while the self-healing capacity of cartilage is very limited due to the lack of vasculature for nutrition supply. Therefore, articular cartilage repair using tissue engineering approach has drawn growing attention [1]. As we know, scaffolds, cells, and growth factors are generally needed for tissue engineering. The interaction between cells and scaffolds plays an important role in controlling cell functions. Many factors can affect the cell-scaffold interaction, such as biological cues and physical cues [2,3]. Biological cues are generally transmitted through the receptors on cell membrane (e.g., integrin) and bioactive molecules in the extracellular matrices, which have been well studied [4,5]. The influence of physical cues, such as surface charges, topography, and mechanical properties, on cell functions has also been extensively investigated [6,7,8,9]. As one of the physical cues, matrix stiffness has been reported to affect cell behaviors, such as cell spreading, migration, proliferation, and differentiation [10,11,12,13]. The Young’s modulus of native tissue is different depending on the tissue type [14]. Therefore, the matrix stiffness needs to be optimized for different cell types and the cellular response to matrix stiffness should be well studied. The fates of some types of cells, such as fibroblasts [15], neutrophils [16], and mesenchymal stem cells (MSCs) [17], have been found to be affected by matrix stiffness. For chondrocytes, it has been reported that substrates with a low stiffness can help to maintain their chondrogenic phenotype due to the round morphology of chondrocytes [18]. However the result is based on 2D cell culture on hydrogel surface that cannot well mimic the 3D mechanical microenvironment in native cartilage. Therefore, it is necessary to study the optimal stiffness of matrix for maintaining chondrogenic phenotype in a 3D culture platform. 

A large variety of synthetic or natural materials have been used to fabricate hydrogels with controlled stiffness. For example, polyethylene glycol (PEG) hydrogels with Young’s modulus from 34–1370 kPa have been synthesized through photo-polymerizing by using different concentrations of macromer [19]. Arginine-glycine-aspartic acid (RGD) peptide incorporating polyethylene glycol dimethacrylate (PEGDM) hydrogels with Young’s modulus from 2–6 kPa have been used to study the chondrocyte extracellular matrix formation and phenotype maintenance [20]. Hydrogels of hyaluronic acid (HA) [21], agarose [22], and alginate [23] with gradient Young’s modulus have also been prepared by altering crosslinking density or macromer concentration. Gelatin, as a proteinaceous material, is derived from collagen by hydrolytic degradation [24,25]. Gelatin has similar chemical composition to that of collagen, which makes gelatin to be one of the most useful biomaterials for tissue engineering [26,27,28]. Gelatin hydrogels have been used to investigate the interaction between matrix and cells because of their similarity to the in vivo microenvironments [29,30].

To prepare gelatin hydrogels, photo-crosslinking method has been proposed because of the advantages of injectability, mild crosslinking condition, and low cytotoxicity [31,32]. Furthermore, the stiffness of photo-crosslinked gelatin hydrogels can be controlled by different degree of crosslinking. Introduction of methacryloyl group in gelatin molecules is a frequently used method to prepare photo-reactive gelatin derivatives. Different strategies have been used to adjust the stiffness of gelatin methacryloyl (GelMA) hydrogels. It has been reported that the stiffness of GelMA hydrogels can be varied from 5–20 kPa by blending GelMA with other polymers such as alginate and HA [33]. Variation of GelMA concentration can also result in different stiffness of GelMA hydrogels [28,34]. However, in these cases, it is difficult to separate the influence from stiffness and other factors, such as composition and concentration. Therefore, it is desirable to prepare hydrogels with different stiffness while keeping their composition and matrix concentration at the same level. Such hydrogels may separate the influence of stiffness from that of other factors, such as composition, matrix concentration and adhesion site density.

In this study, the influence of matrix stiffness on the maintenance of chondrocyte phenotype was investigated by using a series of GelMA hydrogels that had different stiffness but the same gelatin concentration. The GelMA hydrogels were prepared by adjusting the degree of GelMA functionalization. Bovine articular chondrocytes were encapsulated in these hydrogels through the photo-crosslinking method and then cultured for 14 days. The behaviors and fates of chondrocytes in the hydrogels were studied by using cytoskeleton staining, ECM secretion assay, histological staining, and gene expression.

## 2. Materials and Methods

### 2.1. Synthesis of GelMA Macromers

GelMA macromers were synthesized according to a previously described method [35]. 5 g gelatin (type A, 300 bloom, Sigma-Aldrich, St. Louis, MO, USA) was dissolved in 45 mL of phosphate-buffered saline (PBS) at 60 °C under stirring to obtain a 10 wt % gelatin aqueous solution. For the synthesis of GelMA macromer with high degree of functionalization (DoF), the pH of buffer solution was adjusted to 7.6 with 1 N sodium hydroxide solution (Wako, Osaka, Japan) before gelatin dissolution. Different volumes of methacrylic anhydride (denoted as MA, Sigma-Aldrich, St. Louis, MO, USA) (0.2 mL, 1 mL, and 5 mL) was added into the gelatin solution at a rate of 0.5 mL/min under stirring at 50 °C to prepare GelMA with different introduction ratio of MA. After reaction in dark for 3 h, the products were diluted with five-fold warm PBS (50 °C) and then dialyzed against Milli-Q water for seven days at 40 °C using a dialysis membrane (12–14 kD molecular weight cut-off, Spectrum Laboratories Inc., Rancho Dominguez, CA, USA) to remove salts and excess free MA. After that, the products were lyophilized for two days to obtain white porous foam and stored at −20 °C for further use.

### 2.2. ^1^H Nuclear Magnetic Resonance (NMR)

The degree of methacryloyl functionalization was quantified by using ^1^H NMR according to a previously described method [36]. ^1^H NMR spectra were collected by using a NMR spectrometer (JEOL Co. Ltd., Tokyo, Japan) with a single axis gradient inverse probe at a frequency of 300 MHz. Before the measurement, 20 mg of GelMA macromers was completely dissolved in 1 mL deuterium oxide containing 0.05 wt % 3-(trimethylsilyl)propionic-2,2,3,3-d_4_ acid sodium salt for calibration (Sigma-Aldrich, St. Louis, MO, USA). The gelatin without functionalization was also examined for calculating the degree of methacryloyl substitution using the following Equation (1):

DoF = 1 − (lysine methylene proton of GelMA)/(lysine methylene proton of Gelatin) × 100%(1)

### 2.3. RGD Density Measurement

The RGD density in GelMA macromers was quantified by reacting arginine groups with 9,10-phenanthrenequinone (Wako) to produce a fluorescence compound. Briefly, 1 mg/mL gelation or GelMA was mixed with 300 μL of an ethanol solution of 9,10-phenanthrenequinone (150 μM) and 50 μL of NaOH aqueous solution (2 N). The mixture was incubated at 60 °C under dark for 3 h. Then 200 μL of each sample was mixed with 200 μL of HCl (1.2 N) and the mixture was allowed to stand at room temperature under dark for 1 h. The emission of the mixture was measured at an excitation wavelength of 312 nm by an FP-6500 spectrofluorometer (JASCO, Tokyo, Japan) [37,38]. 

### 2.4. Preparation of GelMA Hydrogels

GelMA macromers (10 wt %) and photo-initiator, 2-hydroxy-1-(4-(hydroxyethoxy) phenyl)-2-methyl-1-propanone (Irgacure 2959, Sigma-Aldrich, St. Louis, MO, USA) (0.5 wt %), were dissolved in PBS at 50 °C. The solution was added into the space between two quartz glass coverslips separated by a 1 mm-thick spacer made from silicone gel sheet (KOKEN, Tokyo, Japan). The construct was exposed to 365 nm UV light (CL-1000, Funakoshi, Tokyo, Japan) at a distance of 20 cm for 2 min.

### 2.5. Mechanical Testing 

Atomic force microscopy (AFM, MFP-3D-Bio; Asylum Research, Santa Barbara, CA, USA) was applied to determine the stiffness of GelMA hydrogels. The hydrogel samples were incubated in PBS at 37 °C for 24 h and then their stiffness was measured at room temperature. An optical microscope was used to control the position of the AFM tip. Silicon nitride cantilevers (Bruker, Camarillo, CA, USA) with a 600 nm diameter glass ball were used as the probe. The exact spring constant was measured before each experiment using a thermal tuning method. The force curves were collected and fitted to Hertz’s contact model to calculate the Young’s modulus [8]. Nine samples of each group were measured for calculation of means and standard deviations.

### 2.6. Swelling Ratio Measurement

To determine the mass swelling ratio, hydrogels were punched into disks with a 6 mm biopsy punch. The hydrogel disks were soaked in PBS at 37 °C for 24 h. The samples were blotted with a KimWipe paper to remove the residual liquid and weighed to obtain the equilibrium wet weight. The dry weight was the sample weight after freeze-drying. The mass swelling ratio was calculated by dividing the equilibrium wet weight by the dry weight of the hydrogel disks [34]. 

### 2.7. Measurement of Enzymatic Degradation of Hydrogels

GelMA hydrogels with different stiffness were prepared and punched into disk with an 8 mm diameter biopsy punch following by swollen in PBS for 24 h to reach swelling equilibrium. The swollen hydrogel disks were immersed in 2 mL of PBS containing 10 units·mL^−1^ of collagenase (Worthington Biochemical Corp., Lakewood, NJ, USA) and incubated at 37 °C in an orbital shaker at a shaking speed of 60 rpm. At the time points of 1, 2, 4, 9, and 20 h, the hydrogel samples were taken out and weighed after being blotted. The degradation degree of the hydrogels was determined by normalizing the residual hydrogel wet weight to the initial wet weight. Three samples were used at every time point for the measurement to calculate means and standard deviations.

### 2.8. Culture of Chondrocytes in Hydrogels 

Chondrocytes were isolated from articular cartilage from the knees of a nine week old calf according to previously reported protocol [39,40]. The isolated primary chondrocytes were cultured in 75 cm^2^ tissue culture flasks in Dulbecco’s Modified Eagle Medium (DMEM) supplemented with 10% fetal bovine serum, 4500 mg·L^−1^ glucose, 4 mM glutamine, 100 U·mL^−1^ penicillin, 100 µg·mL^−1^ streptomycin, 0.1 mM nonessential amino acids, 0.4 mM proline, 1 mM sodium pyruvate, and 50 µg·mL^−1^ ascorbic acid at 37 °C and 5% CO_2_. The cells were subcultured after confluence and the cell culture medium was refreshed every three days. The chondrocytes at passage two were used in the following experiments. The cells were detached with a trypsin/EDTA solution, collected by centrifugation, counted with a hemocytometer and re-suspended in the PBS solutions containing GelMA macromers (10 wt %) and photo-initiator Irgacure 2959 (0.5 wt %) to obtain cell suspension at a density of 2 × 10^7^ cell/mL. The suspension solution was used to prepare cell-laden hydrogels in the same manner as above described. The quartz glass coverslips were sterilized before usage and all of the experimental procedures were conducted sterilely in a clean bench. The cell-laden hydrogels were then punched into disks (6 mm diameter × 1 mm thickness) with a sterile 6 mm biopsy punch. The cells in the hydrogel disks were cultured in DMEM medium at 37 °C and 5% CO_2_ under shaking for 14 days and the medium was changed every two days. 

### 2.9. Cell Viability Assay

Live/dead staining was performed to evaluate cell viability of chondrocytes in the hydrogels by using Cellstain Double Staining Kit (Dojindo Laboratories, Tokyo, Japan). After one and seven days of culture, the cell-laden hydrogel disks were washed with PBS and incubated with serum-free medium containing calcein-AM (2 µM) and propidium iodide (4 µM) for 15 min. After that, the live cells and dead cells in the interior areas of hydrogels were observed under a fluorescence microscope (U-RFL-T; Olympus, Tokyo, Japan).

### 2.10. Cytoskeleton Actin Filament Staining

The cytoskeleton of chondrocytes in the hydrogels after seven and 14 days of culture was observed by actin filament (F-actin) staining. The cell-laden hydrogel disks were washed with PBS three times, fixed with 4% paraformaldehyde at 4 °C for 24 h, and further washed two times with PBS. And then the cell-laden hydrogel disks were immersed in 5 mL of 0.2% Triton X-100 for 50 min to permeabilize the cells. After being washed with PBS three times and blocked with 1% bovine serum albumin (BSA) solution at room temperature for 30 min, the samples were immersed in 1 mL PBS containing 40-fold diluted Alexa FluorVR 488 phalloidin (Invitrogen, Carlsbad, CA, USA) for 60 min to stain actin filaments. Cell nuclei were stained with 1000-fold diluted 4′,6-diamidino-2-phenylindole dihydrochloride (DAPI, Dojindo Laboratories, Kumamoto, Japan) solution in PBS at room temperature for 10 min. After staining, cell-laden hydrogel disks were rinsed with PBS again and their fluorescence images were captured using a confocal microscope (LSM 510 Meta; Zeiss, Oberkochen, Germany).

### 2.11. Histological Stainings

After 14 days of culture, the cell-laden hydrogel constructs were washed with PBS and fixed with 10% neutral buffer formalin at room temperature for two days. After that, the samples were dehydrated in a series of ethanol solution with increasing ethanol concentration from 70%–99.5%, embedded in paraffin and sectioned to obtain cross-sections having 5 µm thickness. The cross-sections were deparaffinized and stained with hematoxylin and eosin dyes (HE staining) for cell morphology and safranin-O dye for glycosaminoglycan. The stained samples were observed under an optical microscope (IX-71; Olympus, Tokyo, Japan).

### 2.12. Quantification of DNA and Sulfated Glycosaminoglycan (sGAG)

The DNA amount in each hydrogel was quantified to evaluate the proliferation of chondrocytes. The cell-laden hydrogels with different stiffness were harvested and freeze-dried after being cultured for 14 days. The freeze-dried cell-laden constructs were digested by 500 µL papain solution (Sigma-Aldrich, St. Louis, MO, USA) prepared by dissolving papain at a concentration of 400 mg·mL^−1^ in 0.1 M phosphate buffer (pH 6.0) containing 5 mM cysteine hydrochloride and 5 mM ethylenediaminetetraacetic acid (EDTA). 10 µL of papain digestion solution was used to measure the DNA content by using Hoechst 33258 dye (Sigma-Aldrich, St. Louis, MO, USA) to produce fluorescence. The fluorescence intensity was read with an FP-6500 spectrofluorometer (JASCO, Tokyo, Japan) at an excitation/emission wavelength of 360 nm and 460 nm. The sGAG content in each digestion solution was measured by using Blyscan™ Glycosaminoglycan Assay Kit (Biocolor, County Antrim, UK) according to the instruction offered by the product. Three samples in each group were used for the measurement to calculate means and standard deviations.

### 2.13. Real-Time PCR Analysis

Expression of genes encoding collagen type II and aggrecan in the chondrocytes cultured in the different stiffness hydrogels was analyzed by a real-time polymerase chain reaction (real-time PCR) [41]. After being cultured for 14 days, the cell-laden hydrogel samples were washed with PBS three times, frozen in liquid nitrogen, and crushed into powder by an electric crusher. The powder samples were dissolved in Sepasol solution (1 mL per sample) to isolate the contained RNA. The RNA was converted to cDNA by MuLV reverse transcriptase (Applied Biosystems, Framingham, MA, USA). Real-time PCR was used to amplify glyceraldehyde-3-phosphate dehydrogenase (Gapdh), type II collagen (Col2a1) and aggrecan (Acan). The reactions were run for 40 cycles using a 7500 real-time PCR System (Applied Biosystems, Ghent, Belgium). The expression level of *GAPDH*, a housekeeping gene, was used as an endogenous control. The expression level of the target gene was then calculated using the 2^−ΔΔ*C*t^ formula with reference to the chondrocytes from 2D expansion culture. The primer and probe sequences were chosen according to previous study [36]. Three samples in each group were used for the measurement to calculate means and standard deviations.

### 2.14. Statistical Analysis

All data were reported as the mean ± standard deviation (SD). Statistical analysis was performed using a one-way analysis of variance and Tukey’s post hoc test for multiple comparisons. All statistical analyses were executed using KyPlot 2.0 beta 15 (Koichi Yoshioka, 1997**–**2001).

## 3. Results and Discussion

### 3.1. Synthesis and Characterization of GelMA Macromers

**Figure 1:**
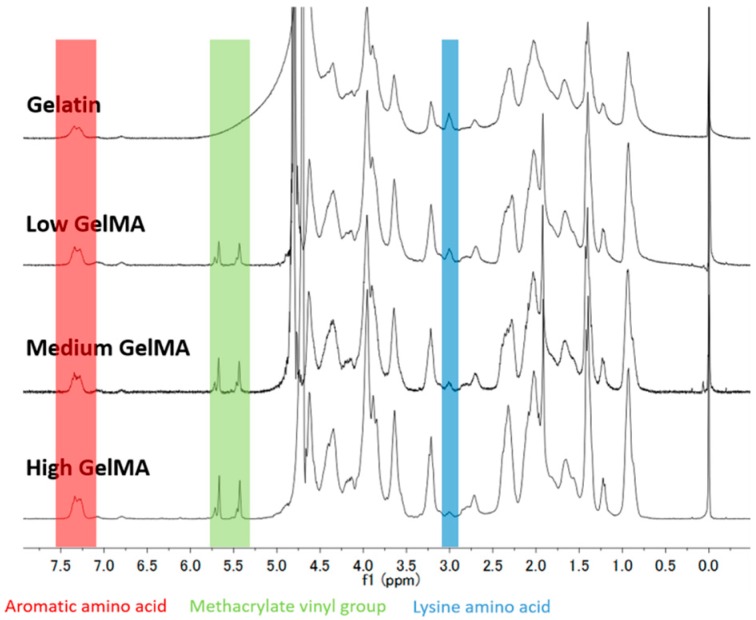
^1^H NMR spectra of unmodified gelatin (Gel), GelMA macromers with different DoF (Low GelMA, Medium GelMA, and High GelMA).

The GelMA macromers with three different degree of functionalization were synthesized by adjusting the volume of methacrylic anhydride (0.2 mL, 1.0 mL, and 5.0 mL per 5 g gelatin) during the reaction. The conjugation of methacryloyl groups to gelatin molecules was confirmed by the ^1^H NMR spectra (Figure 1). The increase of signal at δ = 5.4 and 5.7 ppm (the protons of methacrylate vinyl group of MA) and decrease of signal at δ = 2.9 ppm (the protons of methylene of lysine signal) confirmed the increase of modification degree with the feed MA amount. Since the proton signal of the aromatic amino acid moieties in gelatin remained constant, their intensity was used to normalize the intensity of other protons in different samples. Therefore, the DoF was calculated by comparing the proton signal at δ = 2.9 ppm of unmodified gelatin and GelMA. The DoF of the three types of GelMA macromers increased from 25.8% ± 0.7% to 91.7% ± 1.4% by adjusting the feed ratio of MA to gelatin (Table 1).

### 3.2. RGD Density in Gelatin and GelMA Macromers

**Figure 2:**
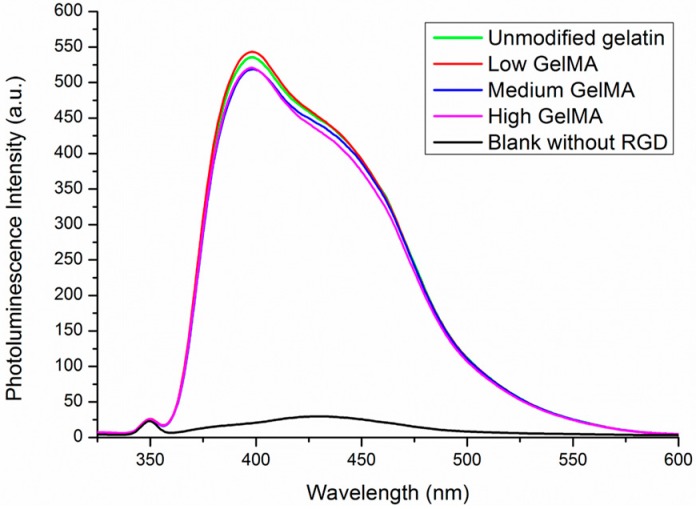
Photoluminescence spectra of 9,10-phenanthrenequinone after reaction with gelatin and GelMA macromers.

The arginine residues in gelatin also have amino groups that may react with MA. Therefore, the content of arginine residues in the GelMA macromers was measured to confirm if the RGD density was changed after the modification. The amino groups in arginine residues can react with 9,10-phenanthrenequinone to produce a fluorescence compound. Unmodified gelatin and the three types of GelMA macromaers with low, medium, and high DoF had almost the same level of fluorescence absorbance (Figure 2). Blank control without RGD prepared by deionized water did not show obvious fluorescence absorbance at the wavelength range. The results indicated that the arginine content in Gel and GelMA macromers was almost the same, which suggests that the arginine should be not involved in the reaction even at a high DoF. Therefore, the RGD density in all the GelMA hydrogels prepared from the three types of GelMA macromers should be kept at the same levels, which was important to exclude the influence of different density of active RGD motifs.

### 3.3. Young’s Modulus and Swelling Ratio of GelMA Hydrogels

**Figure 3:**
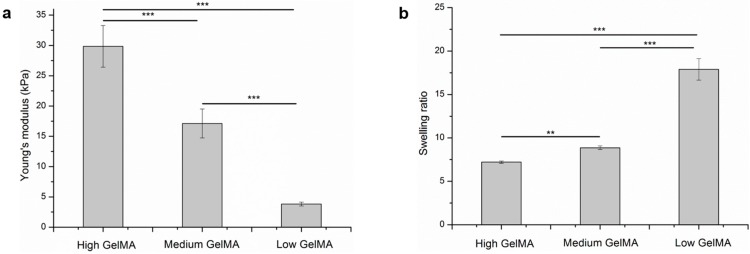
Young’s modulus (**a**) and equilibrium swelling ratio (**b**) of GelMA hydrogels with high, medium, and low stiffness. Means ± SD, *n* = 3. *** *p* < 0.001; ** *p* < 0.01.

Three types of hydrogels were prepared from GelMA macromers with the same concentration of gelatin (10 wt %) but different DoF. The stiffness of GelMA hydrogels was measured by AFM. The GelMA hydrogels prepared from GelMA with low, medium, and high DoF had Young’s modulus of 3.8 ± 0.3, 17.1 ± 2.4, and 29.9 ± 3.4 kPa, respectively (Figure 3a). As expected, high DoF resulted in GelMA hydrogels with significantly increased Young’s modulus. The results showed that the GelMA hydrogels with a broad range of stiffness could be generated by adjusting the DoF of macromers. 

Swelling ratio of hydrogels is an important factor that should be considered for tissue engineering application. The swelling ratio of GelMA hydrogels with low, medium, and high stiffness was calculated to be 17.89 ± 1.24, 8.86 ± 0.20, and 7.20 ± 0.12, respectively (Figure 3b). Clearly, the swelling ratio of hydrogels decreased with the increase of stiffness. This may be because the high DoF of methacryloyl in high stiffness GelMA hydrogel could increase the density of cross-linking network. The high cross-linking density could restrain the swelling of the hydrogels and resulted in a low swelling ratio.

### 3.4. Enzymatic Degradation of GelMA Hydrogels

**Figure 4:**
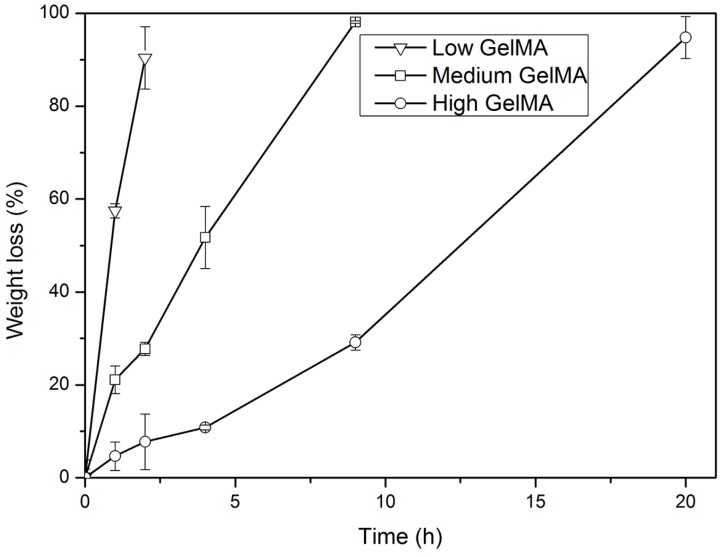
Enzymatic degradation of GelMA hydrogels in the presence of 10 unit·mL^−1^ of collagenase at 37 °C. Means ± SD, *n* = 3.

It is necessary to investigate the biodegradability of hydrogels before their biomedical applications. The enzymatic degradability of GelMA hydrogels with different stiffness was examined in the presence of collagenase. Collagenase is a member of the matrix metalloproteinase (MMP) family that can degrade and remodel the extracellular matrix for cell spreading and migration [42]. Herein, collagenase was used because it can accelerate the degradation of proteolysis-sensitive gelatin hydrogels. The weight loss rate of low, medium, and high stiffness GelMA hydrogels after treatment with collagenase is shown in Figure 4. GelMA hydrogels with higher stiffness were degraded more slowly when compared to those with lower stiffness. These results suggested that the enzymatic degradability of GelMA hydrogels could be controlled by the degree of cross-linking and hydrogel stiffness. Higher cross-linking degree resulted in higher stiffness and slower degradation.

### 3.5. Cell Viability in GelMA Hydrogels

**Figure 5:**
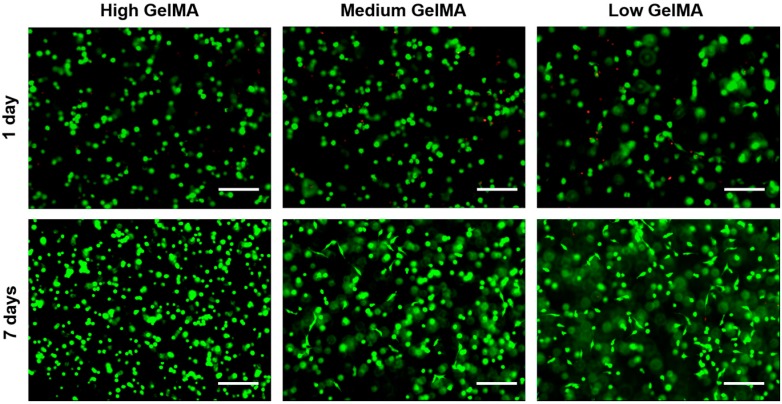
Live/dead staining of chondrocytes cultured in the GelMA hydrogels for one day and seven days. Scale bar = 100 µm. (Green: live cells; Red: dead cells).

Cell viability was investigated by live/dead staining after chondrocytes were encapsulated in the GelMA hydrogels and cultured for one and seven days. In all the groups, most of the chondrocytes were alive (green color) and only few dead cells were observed (red color) after one and seven days of culture (Figure 5). The results indicated that the cells in the hydrogels had high cell viability even after the photo-crosslinking process, suggesting that it may be a good strategy to use the GelMA hydrogels for cell encapsulation. Furthermore, the live cell density seemed to be the same for all the GelMA hydrogels, suggesting that the stiffness of GelMA did not have obvious influence on cell viability.

### 3.6. Cytoskeleton Organization in GelMA Hydrogels

**Figure 6:**
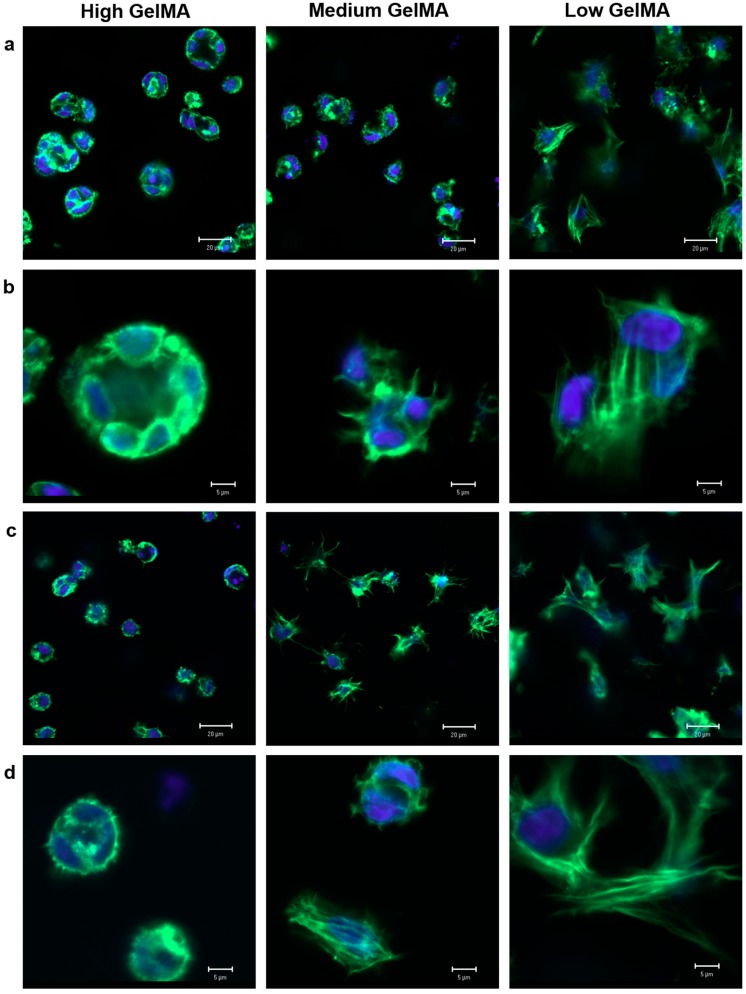
Confocal laser microscopy images of chondrocytes that were stained with F-actin (green) and cell nuclei (blue) after being cultured in GelMA hydrogels for seven days (**a**,**b**) and 14 days (**c**,**d**). (**a**,**c**) scale bar = 20 µm; (**b**,**d**) scale bar = 5 µm.

Immunofluorescence staining of F-actin was performed to visualize the cellular morphology and cytoskeleton organization in the hydrogels (Figure 6). Chondrocytes in the high stiffness GelMA hydrogels had almost round shape and no obvious F-actin stretch, which indicated that the cells did not spread well after being cultured in the high stiffness hydrogels for seven and 14 days. Furthermore, some cell clusters were observed in the high stiffness GelMA hydrogels. In contrast, the cell elongated in the low stiffness GelMA hydrogels with abundant F-actin filaments after culture for seven and 14 days. Chondrocytes cultured in the medium stiffness GelMA hydrogels showed round morphology after 7 days of culture and spreading morphology after 14 days of culture. The results indicated that cell morphology could be controlled by the stiffness of hydrogels.

Cytoskeletal configuration and regulation have been reported to play an important role in the process of chondrogenesis [43]. It was recognized that round cell morphology and decreased actin cytoskeletal organization are beneficial for the chondrogenesis of mesenchymal stem cells (MSCs) [43,44]. Spherical aggregation was also beneficial for chondrogenesis due to the increased intercellular contacts [45,46]. The high stiffness GelMA hydrogels had a high Young’s modulus and a high degree of cross-linking, which inhibited cell spreading and resulted in weak F-actin filament network formation. The microenvironment of high stiffness hydrogels also promoted spherical aggregation. Similar cell morphology was observed in medium stiffness GelMA hydrogels after seven days of culture. However, most cells in the medium stiffness GelMA hydrogels spread after 14 days of culture, which might be due to the continuous proteolytic degradation of hydrogels. This suggested the hydrogels with high stiffness could support the maintenance of the round morphology of chondrocytes, in agreement with the previous study [47].

### 3.7. Histological Stainings

**Figure 7:**
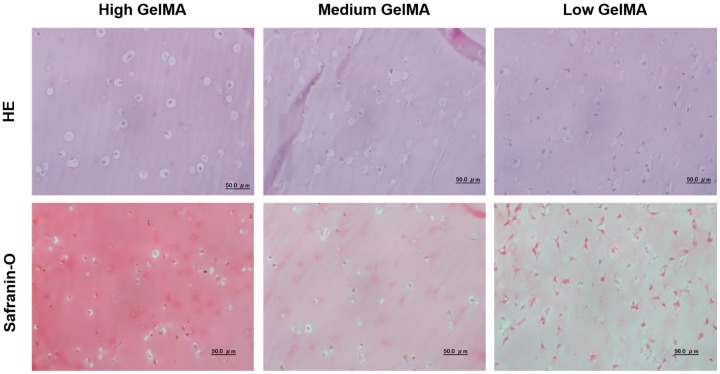
HE and safranin-O stainings of chondrocytes cultured in the GelMA hydrogels for 14 days.

Cell morphology and cartilaginous matrices production of the chondrocytes after 14 days of culture in the GelMA hydrogels were further investigated by histological staining (Figure 7). HE staining indicated a homogeneous cell distribution for all the GelMA hydrogels. The chondrocytes showed round morphology in the high stiffness GelMA hydrogels, while showing a partially spreading morphology in medium stiffness GelMA hydrogels. In contrast, cells in the low stiffness GelMA hydrogels showed an elongated morphology. The elongation of cells in low stiffness hydrogels might be a signal of losing the chondrogenic phenotype [43,44]. Safranin-O staining indicated that more proteoglycans were detected in the high stiffness GelMA hydrogels than the other two groups. The cells cultured in medium stiffness GelMA hydrogels showed partially positive staining of proteoglycans, while the chondrocytes cultured in the low stiffness GelMA hydrogels lost their proteoglycans secretion capacity obviously.

One reason for these results may be that high stiffness offered a better stimulus to chondrocyte through the pathways to maintain their chondrogenic phenotype in 3D culture [44,48,49]. Another reason may be that the GelMA hydrogels with high stiffness could promote the chondrocytes cluster formation. The chondrocytes aggregation has been reported to facilitate the outcome of cartilage repair [50,51], because cell-cell interaction plays a critical role in chondrogenesis [52,53,54]. The staining results indicated that GelMA hydrogels with high stiffness could support the chondrogenic phenotype.

### 3.8. Quantification of DNA, Cartilaginous Matrices, and Gene Expression

**Figure 8:**
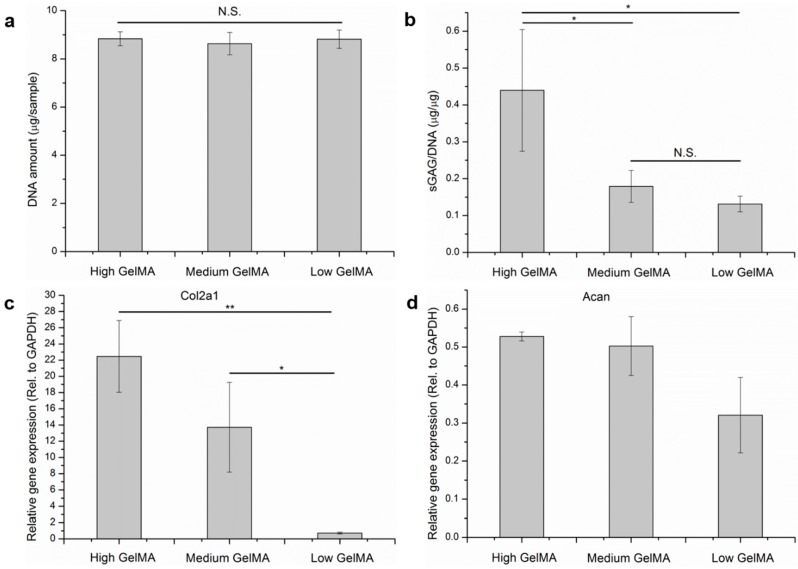
DNA amount (**a**); sGAG/DNA ratio (**b**); and expression of genes encoding collagen type II (**c**) and aggrecan (**d**) of chondrocytes cultured in the GelMA hydrogels for 14 days. Means ± SD, *n* = 3. ** *p* < 0.01; * *p* < 0.05.

To investigate whether the stiffness affect cell proliferation, DNA amount measurement was performed after the cells were cultured in the GelMA hydrogels for 14 days (Figure 8a). The cells cultured in different GelMA hydrogels showed almost the same DNA amount, indicating the stiffness of hydrogels had no obvious influence on cell proliferation.

The secretion of ECM proteins is very important for cartilage regeneration because chondrocytes in cartilage are surrounded by abundant ECMs, such as sGAG. The sGAG/DNA ratio was calculated from the amount of sGAG and DNA to show the production capacity of cartilaginous ECM by each cell (Figure 8b). The sGAG/DNA ratio of chondrocytes in high stiffness GelMA hydrogels was significantly higher than the other groups after 14 days of culture. No significant difference could be seen in the medium and low stiffness GelMA hydrogels.

The expression of genes encoding the two typical cartilaginous matrices (collagen type II and aggrecan) was analyzed by real-time PCR (Figure 8c,d). Chondrocytes cultured in high stiffness GelMA hydrogels showed the highest expression level of collagen type-II and aggrecan genes after 14 days of culture. The quantification data of cartilaginous matrices and their gene expression were in good agreement with the staining results, indicating that chondrocytes cultured in the high stiffness GelMA hydrogels had the best maintenance capacity of chondrogenic phenotype. 

The rapid degradation and stress relaxation characters of low stiffness GelMA hydrogels may promote the dedifferentiation of chondrocytes as they down-regulated the gene expression of collagen type II and aggrecan. It has been reported that high spreading can be observed in physiological extracellular matrices with high degradation rate and quick stress relaxation [55,56]. All of our results suggested the GelMA hydrogels with high stiffness had the best function for the maintenance of chondrocyte phenotype.

The results of this study are different from some previous studies [19,20,22]. Some previous studies have reported that lower stiffness hydrogels had a better maintenance function of chondrogenic phenotype. The difference of stiffness range and material properties between this study and the previous studies might be the reasons for the different results. The high stiffness in this study was at the same range as the low stiffness of the previous studies. The Young’s modulus of hydrogels in this study was controlled to mimic the mechanical property of cartilage ECM [57,58,59]. However, the stiffness range was far from the rigidity of native cartilage (400–800 kPa) [59,60]. This might be one reason. The materials property including adhesion sites and degradable sites might be another reason. In PEG, agarose and HA hydrogels, cells keep round shape in lower stiffness hydrogels until sufficient extracellular matrices are produced [61,62]. But in gelatin based hydrogels, cells can easily spread [55]. Presence of RGD in the hydrogels can promote cell spreading. It is important to investigate the influence of matrix stiffness on chondrogenic phenotype by using natural ECM protein materials (collagen and gelatin, etc.) because they have RGD and MMP degradable sequences to better mimic the microenvironment as compared with other natural polymer or synthetic materials, such as HA and PEG.

## 4. Conclusions

GelMA hydrogels with different stiffness and the same RGD density were fabricated by adjusting the degree of methacryloyl substitution of same concentration of gelatin. The Young’s modulus increased with the degree of functionalization. Cell cultured in the hydrogels showed high viability. Bovine chondrocytes cultured in the high stiffness GelMA hydrogels showed rounder morphology, secreted more cartilaginous matrices, and exhibited higher expression of collagen II and aggrecan genes compared to the cells cultured in medium and low stiffness GelMA hydrogels. The high-stiffness GelMA hydrogels had the highest capacity of maintaining the phenotype of chondrocytes.

## Figures and Tables

**Table 1:** The DoF of Low GelMA, Medium GelMA, and High GelMA.

Sample	Feed Ratio of Gel (g)/MA (mL)	DoF (%)
Low GelMA	5/0.2	25.8 ± 0.7
Medium GelMA	5/1.0	52.5 ± 1.2
High GelMA	5/5.0	91.7 ± 1.4
